# Molar Uprighting Using Segmental Wiring Technique (M.U.S.T.): A Case Report

**DOI:** 10.7759/cureus.88166

**Published:** 2025-07-17

**Authors:** Rashika Singhania, Akansha Thapliyal, Tanu Nangia, Carrolene Langpoklakpam, Sakshi Shah

**Affiliations:** 1 Pediatric and Preventive Dentistry, Manav Rachna Dental College, School of Dental Sciences (SDS) Manav Rachna International Institute of Research and Studies (MRIIRS), Faridabad, IND

**Keywords:** bracket, double tubes, flexible archwires, molar uprighting, m.u.s.t. technique

## Abstract

A tipped molar is a relatively common condition that can compromise oral health by increasing the risk of periodontal defects, complicating prosthetic restoration, and creating unfavorable occlusal forces. This case report presents a practical clinical approach for molar uprighting using the Molar Uprighting Using Segmental Wiring Technique (M.U.S.T.) in a 12-year-old female patient. In this case, a mesially tipped second molar (tooth 37) impeded the placement of a stainless steel crown (SSC) on tooth 36 following root canal treatment. The technique involves using double buccal tubes bonded to the molars and flexible archwires, allowing for effective uprighting of posterior teeth with minimal intervention and without complex loop designs or technique-sensitive procedures. Compared to traditional full-fixed appliances, M.U.S.T. offers improved patient comfort and facilitates easier oral hygiene maintenance.

## Introduction

Permanent mandibular first molars are among the earliest to erupt and remain in the oral cavity for a long duration. Without proper oral hygiene, they are highly vulnerable to caries, often leading to partial or complete crown destruction. This loss may result in rotation or tilting of the second and occasionally third molars [1].

Tilted molars can cause periodontal problems, such as inflammation, angular bone loss, and pocket formation. Severe tilting may lead to over-eruption of the opposing teeth, premature contacts, and occlusal interferences, complicating prosthetic treatment. Molar uprighting is often necessary to enable prosthetic rehabilitation and promote overall oral health restoration [2].

Various orthodontic methods, such as cantilever springs, prefabricated Sander springs, helical uprighting springs, NiTi coil springs, push spring appliances, and removable appliance traction, are commonly employed for molar uprighting [2]. A newer technique involves using temporary anchorage devices (TADs), which, although effective, can be expensive, inventory-intensive, and technique-sensitive [3]. In this case report, a simple yet efficient approach, Molar Uprighting Using Segmental Wiring Technique (M.U.S.T.), by Mansour et al. [3] was utilized to upright a tilted mandibular second molar, enabling restoration of the first molar.

## Case presentation

**Figure 1:**
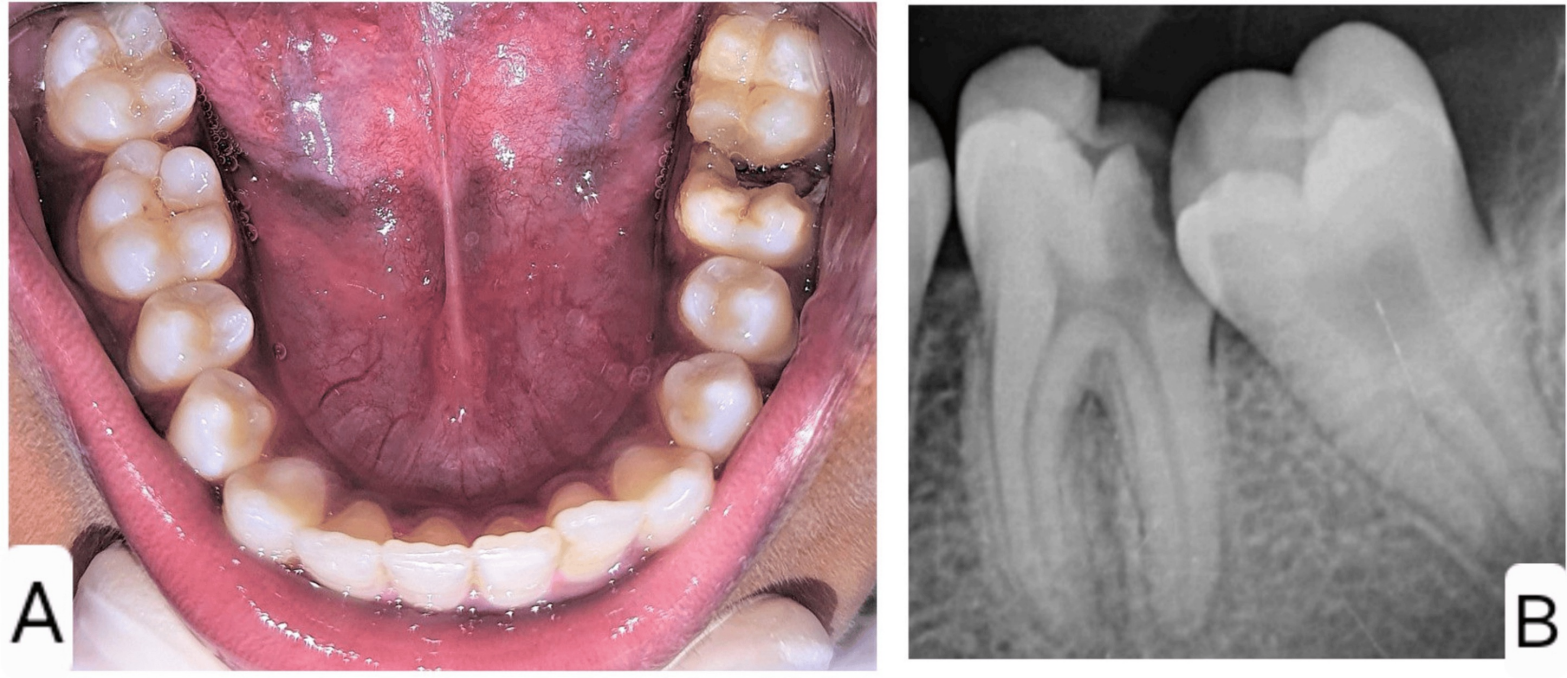
Preoperative photograph (A) and radiograph (B) of the left mandibular permanent first molar showing a large proximal carious lesion

A 12-year-old female patient with no significant medical history presented to the Department of Pediatric and Preventive Dentistry with a complaint of pain and decay in the lower left back tooth region for the past two weeks. Clinical and radiographic evaluations revealed a deep proximal carious lesion with pulpal involvement in relation to tooth 36, leading to a diagnosis of symptomatic chronic irreversible pulpitis (Figures 1A, 1B).

**Figure 2:**
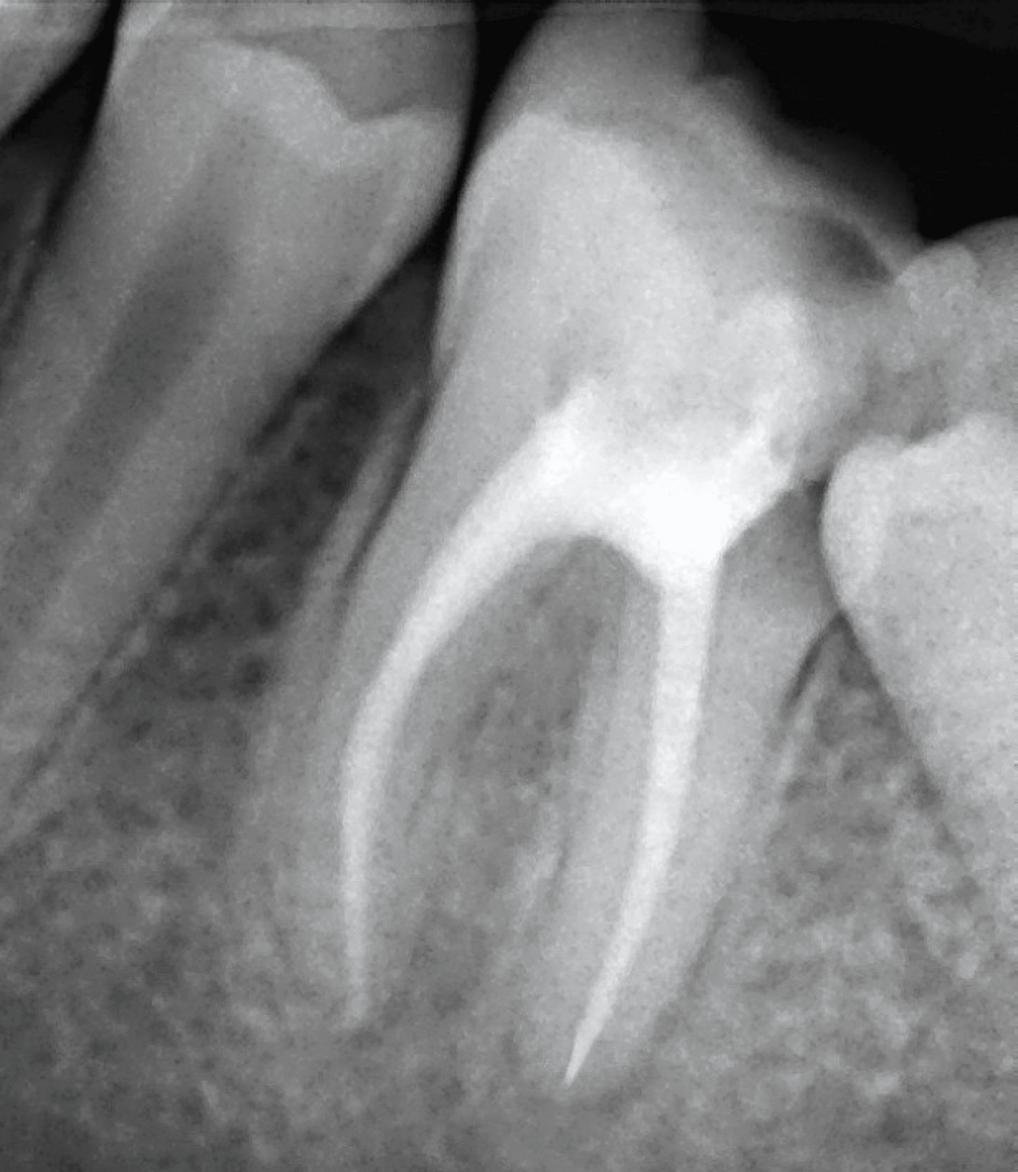
Mesial tilting of the left mandibular permanent second molar, creating a hindrance in the path of placement of the stainless steel crown

The lesion had also resulted in the loss of the distal wall of 36, causing mesial tipping of the adjacent second molar (37), which interfered with the placement of a stainless steel crown (SSC) following root canal treatment (Figure 2).

Root canal therapy was completed for 36, and an SSC was planned as a final restoration.

**Figure 3:**
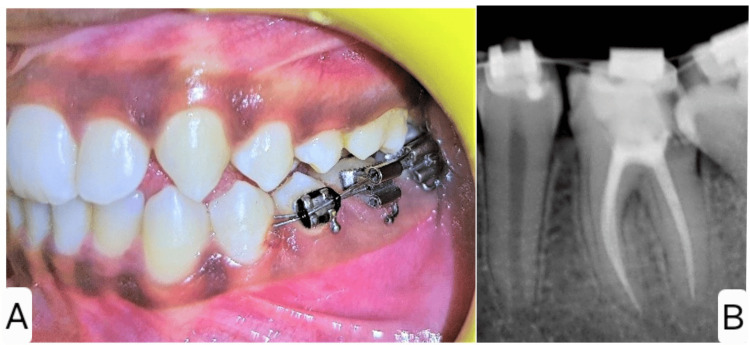
Intraoperative photograph (A) and radiograph (B) of Molar Uprighting Using Segmental Wiring Technique (M.U.S.T.)

To correct the mesial tilting of tooth 37, the M.U.S.T. was planned and implemented. The procedure involved bonding a double buccal tube to the tilted second molar 37 and the first molar 36, along with placing a premolar bracket on the second premolar 35. An initial 0.012-inch super-elastic NiTi archwire was used for uprighting. This wire was threaded through the main tubes of both molars, then looped distally through the auxiliary tubes of the second and first molars, and finally ligated to the premolar bracket (Figures 3A, 3B).

To facilitate proper molar movement, the posterior bite was raised on the opposite (contralateral) side.

**Figure 4:**
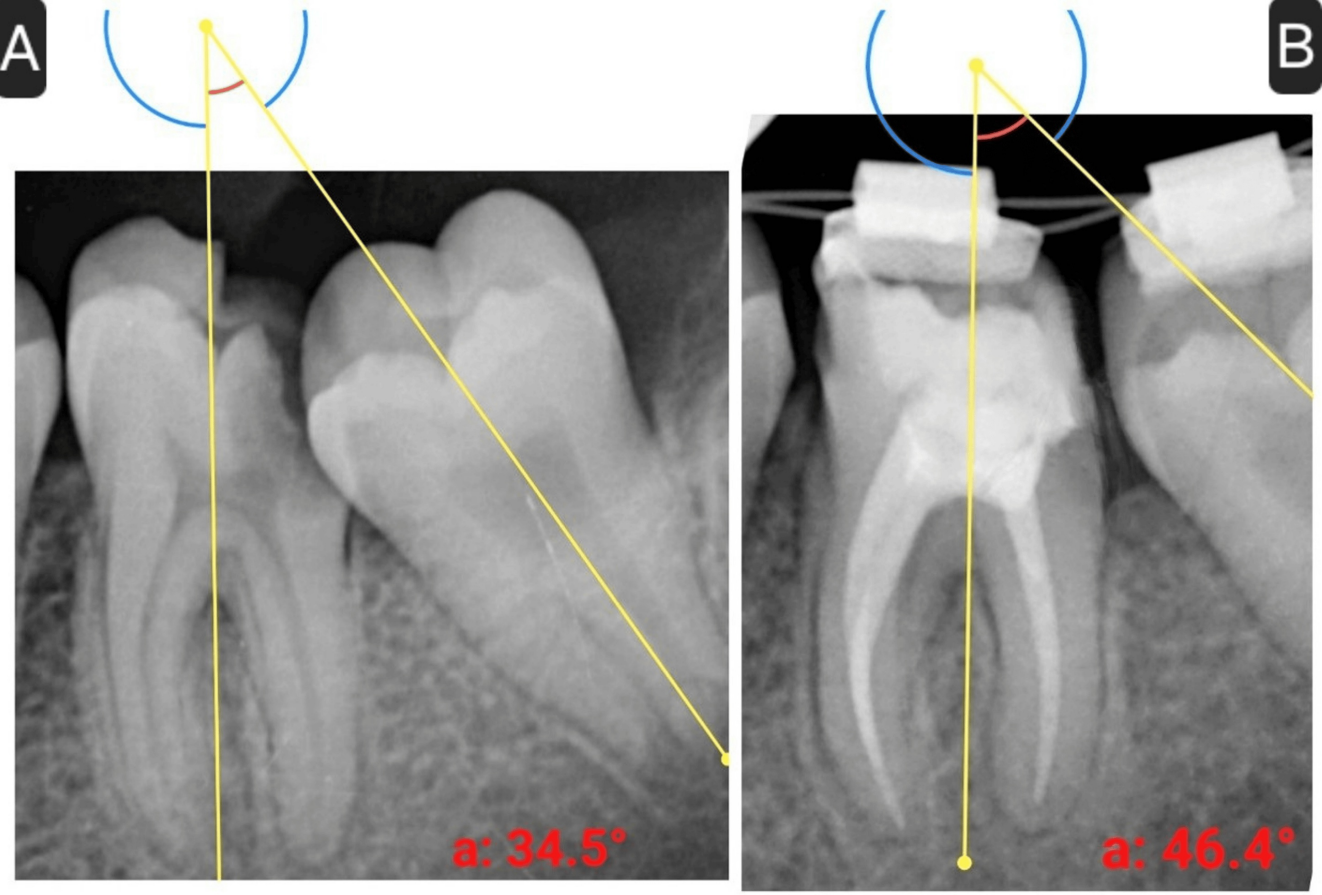
(A) Preoperative angle of inclination of the left mandibular permanent second molar (34.5°). (B) Angle of inclination of the left mandibular permanent second molar following molar uprighting procedure (46.4°)

After two weeks, the wire was replaced with a 0.014-inch NiTi to prevent deformation due to the stress of sharp bends. At the four-week mark, a 0.016-inch wire was introduced. Radiographs were taken at each follow-up to monitor the uprighting progress and assess the inclination of tooth 37. By the third follow-up (at four weeks), radiographic analysis revealed an 11.9° improvement in molar angulation (Figures 4A, 4B).

**Figure 5:**
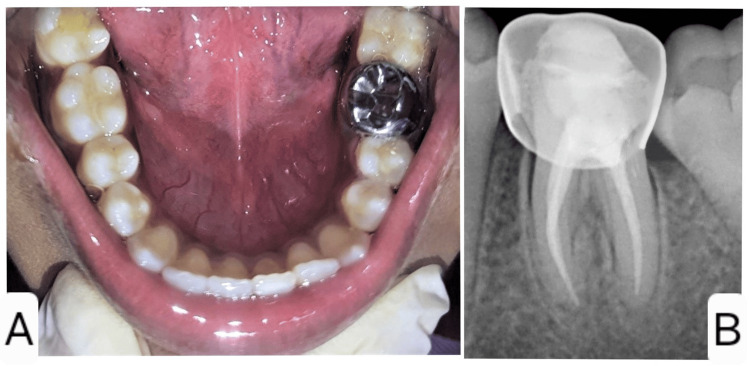
Postoperative photograph (A) and radiograph (B) of the left mandibular permanent first molar following stainless steel crown placement

Although complete uprighting was not achieved due to the patient missing further appointments, an SSC was successfully placed on tooth 36 during this visit (Figures 5A, 5B).

## Discussion

Molar uprighting procedure is recommended as an adjunctive step during restorative and periodontal therapies [4]. Several treatment modalities are mentioned in the literature. These require appliance insertion and application of a precise force for activation that is laborious for the clinician as well as the patient due to complicated appliance designs and restricted intraoral space [5]. Failure in managing the unfavorable reactionary force vectors during the uprighting procedure can cause detrimental effects on areas of the dentition utilized for anchorage [6]. Furthermore, inadequate space around the molars can limit the distal extension of wires, thus causing a reduction in the amount of force generated [5].

Additionally, the absence of a tooth for distal anchorage may complicate the orthodontic procedure for uprighting a tipped last molar, and forces that are generated as a result of the uprighting methodologies, such as extrusive forces on the tilted molars, further impede the mechanics. In this case report, “M.U.S.T.”, as described by Mansour et al. [3], has been used, which allows structured uprighting of tipped molars with minimal observable extrusion and also allows distalization if required. This technique was devised to upright molars without causing extrusion. [4] According to Hsieh et al., the light but steady forces of the super-elastic NiTi wires led to effective uprighting of the tipped molar [4].

The resourcefulness of this technique makes it easy to use in various clinical situations with mesially tipped molars. This technique does not require the use of springs, loops, and TADs, thus greatly lowering both patient discomfort and costs. It even negates the requirement of a distal anchor tooth to accomplish molar uprighting. A limitation of this technique is the arduous work of looping back the wire in the tipped molar’s tubes [3]. However, the lack of occlusal interferences, wire deformations due to mastication, ease of intraoral activation, and relatively less treatment time make this method advantageous [4].

Bae et al. used a small segmental wire with double-sided hooks and elastomeric thread to apply an uprighting force for distal movement of an ectopically erupting molar, emphasizing the ease, effectiveness, and speed of segmental wiring methods [5]. Kim et al. [7] and Jung et al. [8] employed NiTi springs, transferred from model setups with customized resin bases, to achieve the desired tooth movement for space regaining.

In this case, up to 11.9° of uprighting was achieved in four weeks, which created sufficient space for the placement of SSC on the treated molar to reinstate its form and function and avoid relapse of the uprighting achieved. Complete uprighting of 37 could not be achieved in the present case due to the inability of the patient to be present for the entire duration of the treatment.

## Conclusions

M.U.S.T. offers straightforward mechanics for uprighting a mesially tipped molar requiring minimal armamentarium and effort. It maintains patient comfort and presents a cost-effective solution with minimal side effects. However, the outcomes of this report must be interpreted with caution, considering the incomplete treatment and the inherent limitations of a single case report in drawing broader clinical generalizations. Further research is recommended to validate these findings.
